# Chemical Compositions, Antioxidant and Antimicrobial Activities of Essential Oils of *Piper caninum* Blume

**DOI:** 10.3390/ijms12117720

**Published:** 2011-11-08

**Authors:** Wan Mohd Nuzul Hakimi Wan Salleh, Farediah Ahmad, Khong Heng Yen, Hasnah Mohd Sirat

**Affiliations:** 1 Department of Chemistry, Faculty of Science, Universiti Teknologi Malaysia (UTM), Skudai, Johor 81310, Malaysia; E-Mails: nuzoule208@yahoo.com (W.M.N.H.W.S.); hasnah@kimia.fs.utm.my (H.M.S.); 2 School of Chemistry and Environment Studies, Faculty of Applied Sciences, Universiti Teknologi MARA (UiTM) Sarawak, Kota Samarahan, Sarawak 94300, Malaysia; E-Mail: khonghy@sarawak.uitm.edu.my

**Keywords:** essential oil, Piperaceae, *Piper caninum*, antioxidant, antimicrobial

## Abstract

Chemical composition, antioxidant and antimicrobial activities of the fresh leaves and stems oils of *Piper caninum* were investigated. A total of forty eight constituents were identified in the leaves (77.9%) and stems (87.0%) oil which were characterized by high proportions of phenylpropanoid, safrole with 17.1% for leaves and 25.5% for stems oil. Antioxidant activities were evaluated by using β-carotene/linoleic acid bleaching, DPPH radical scavenging and total phenolic content. Stems oil showed the highest inhibitory activity towards lipid peroxidation (114.9 ± 0.9%), compared to BHT (95.5 ± 0.5%), while leaves oil showed significant total phenolic content (27.4 ± 0.5 mg GA/g) equivalent to gallic acid. However, the essential oils showed weak activity towards DPPH free-radical scavenging. Evaluation of antimicrobial activity revealed that both oils exhibited strong activity against all bacteria strains with MIC values in the range 62.5 to 250 μg/mL, but weak activity against fungal strains. These findings suggest that the essential oils can be used as antioxidant and antimicrobial agents for therapeutic, nutraceutical industries and food manufactures.

## 1. Introduction

The genus *Piper* is one of the largest and the most important aromatic and medicinal plants of the Piperaceae family which comprises four genera and approximately 2000 species distributed in the tropical and subtropical regions [1,2]. *Piper* species are used in traditional remedies and folklore medicine all over the world for their antioxidant [3–5], antimicrobial [6–8], anti-inflammatory [9–12] and antifungal [13–15] activities.

*Piper caninum* Bl., locally known as “cabai hutan” or “lada hantu” in Malaysia, is found throughout the tropic mostly in South East Asia such as Thailand and Malaysia. It grows as climber on a small tree in lowland and upland rain forest. The leaves are chartaceous, undersurface glaucous, upper surface green when fresh and turn black when dry. The flowers are very small, about 0.6–0.8 mm diameter, consisting of a petal scale and stamens. The fruits are inflorescence axillary with orange color and have a smell like aniseed. The leaves are used by peoples for chewing. They are added to the betel-quid for treating hoarseness. Besides, it is also used after childbirth [16,17].

To the best of our knowledge, there is no report on the chemical compositions and bioactivity studies of the essential oil of *P. caninum*. So, here we would like to report the chemical composition, antioxidant and antimicrobial activities of the leaves and stems oils of *P. caninum*.

## 2. Results and Discussion

### 2.1. Composition of the Essential Oils

The essential oils, pale yellow color were obtained by hydrodistillation of the leaves and stems of *P. caninum* are presented in Table 1. The chemical compositions of the leaves and stems oils were analyzed by GC, GC-MS and Kovats Indices.

Thirty six components (77.9%) and thirty seven components (87.0%) were identified from the leaves and stems oil, respectively. The leaves oil consists of two phenylpropanoids (18.0%), eleven monoterpenes (29.3%) and nineteen sesquiterpenes (26.7%). The main fraction in the leaves oils were sesquiterpenes hydrocarbons (21.9%) and monoterpene hydrocarbons (21.8%). The most abundant components in the leaves oil were safrole (17.1%), β-pinene (8.9%), linalool (7.0%) and β-caryophyllene (6.7%). In addition, germacrene D (4.9%), α-pinene (4.0%), (*E*)-nerolidol (3.9%), (*Z*)-β-ocimene (3.4%), β-elemene (2.1%) and 2-undecanone (2.0%) were also detected in the leaves oil. On the other hand, the stems oil contains two phenylpropanoids (27.9%), nine monoterpenes (14.7%) and twenty three sesquiterpenes (42.1%). The sesquiterpenes hydrocarbon made up a major fraction in the stems oil which constituted 35.2%. The major components in the stems oils were safrole (25.5%), β-caryophyllene (9.8%), germacrene D (7.8%), β-pinene (4.9%) and δ-elemene (4.1%). Besides, those present in more than 2% were linalool (2.9%), limonene (2.7%), eugenol (2.4%), t-muurolol (2.4%) and bicyclogermacrene (2.3%).

Most of the oil components were similar in both oils. However, camphor (0.3%), α-cubebene (0.5%), eugenol (2.4%), β-cubebene (0.3%), aromadendrene (0.8%), *allo*-aromadendrene (0.3%), α-bisabolene (0.4%), germacrene B (1.1%), globulol (0.3%), t-muurolol (2.4%), α-cadinol (1.0%) and farnesyl acetate (1.2%) were found to be absent in the leaves oil. In addition, myrcene (0.9%), γ-terpinene (0.1%), α-terpinolene (0.4%), 2-nonanone (1.2%), pinocarvone (0.2%), cyclosativene (0.2%), α-ylangene (0.2%), β-bourbonene (1.1%), β-gurjunene (0.2%), (*E*,*E*)-α-farnesene (0.9%), chavibetol (0.9%), elemol (0.2%) and β-eudesmol (0.9%) were absent in the stems oil.

Similar to our results, safrole has been reported to be the major constituents of *Piper auritum* H.B.K. (Panama: leaves oil, 70%) [18], *Piper callosum* Ruiz & Pav. (Brazil: leaves oil, 70%), *Piper hispidinervium* C. DC. (Brazil: leaves oil, 81–88%) [19], *Piper betle* Linn. flowers oil (Taiwan: flowers oil, 27.6%) [20], *Piper mikanianum* Kunth (Brazil: leaves oil, 82%) [21] and *Piper xylosteoides* C. DC. (Brazil: aerial parts, 47.83%) [22]. Safrole is a precursor of many products used as natural insecticides as well as in the perfumery industries [23], while β-caryophyllene is known for its anti-inflammatory and local anaesthetic activities [24]. Since the oils are rich in safrole and β-caryophyllene, leaves and stems oils of *P. caninum* can be used as the source of these components.

### 2.2. Antioxidant Activity

The essential oils were screened for their possible antioxidant activity by β-carotene/linoleic acid, DPPH radical scavenging and total phenolic contents. The results of the antioxidant activity are shown in Table 2.

β-Carotene/linoleic acid bleaching assay was evaluated by measuring the inhibition of conjugated diene hydroperoxides starting from linoleic acid oxidation. The effectiveness of the stems oil (114.9 ± 0.9%) was higher than leaves oil (103.5 ± 0.8%), which were higher compared to BHT (95.5 ± 0.5%). Compounds containing hydrogen atoms in the allylic and/or benzylic positions give better activity in this test because of relatively easy abstraction of hydrogen atom from these functional groups by peroxy radicals formed in the test circumstances [25]. In the stems oil, safrole (25.5%) was present in high amount, which is responsible for the antioxidant activity. The effect of antioxidant on DPPH radical scavenging was thought to be due to their hydrogen donating ability. It has been reported that free radical scavenging activity is greatly influenced by the phenolic components of samples [26]. In this study, the essential oils demonstrated low antioxidant activities, compared to BHT (43.5 ± 0.25 mg/mL) due to low abundance of phenolic components in the essential oils of *P. caninum*.

The amounts of total phenolic in the essential oils were determined spectrometrically according to the Folin-Ciocalteu’s method and calculated as garlic acid equivalents as shown in Table 2. The total phenolic contents of the essential oils are 27.4 ± 0.54 mg GA/g and 19.8 ± 0.42 mg GA/g for leaves and stems oil, respectively. The results indicated that leaves oils gave higher total phenolic than the stems oil, which revealed that there is a relationship between the antioxidant ability and total phenolic contents. The antioxidant activity is mainly due to their redox properties and chemical structure, which can play important role in inhibiting lipoxygenase and free radical scavenging [27].

### 2.3. Antimicrobial Activity

The antimicrobial activity of the essential oil against Gram-positive bacteria (*B. subtilis*, *S. aureus*), Gram-negative bacteria (*P. aeruginosa*, *P. putida*, *E. coli*), yeast (*C. albicans*) and fungi (*A. niger*) was assessed both qualitatively and quantitatively by disc diffusion method and MIC values. Table 3 shows the antimicrobial activity of the essential oil of *P. caninum*.

Both oils showed strong antimicrobial activities against all bacterial strains but moderate to weak activity against fungal strains. The inhibition zones and MIC values for microbial strains, sensitive to the essential oils, were in the range 7–12 mm and 62.5–250 μg/mL, respectively. The leaves oil showed strong MIC value, 62.5 μg/mL towards *E. coli* while the stems oil exhibited MIC value 125 μg/mL against *S. aureus* and *E. coli*. The presence of safrole and β-caryophyllene may contribute to the antimicrobial activity of both oils. Studies on the antimicrobial activities of single aroma compounds found that safrole [28] inhibited the Gram negative, *E. coli* while β-caryophyllene [29] active against the Gram positive bacterium (*S. aureus and E. fecalis*). The stems oil showed moderate antimicrobial activity against *C. albicans* and *A. niger* with MIC value 500 μg/mL, however the leaves oil showed weak activity in both disc diffusion and micro-well dilution methods. Our findings revealed that the sensitivity of the bacterial strains to the leaves oil decreases in the order: *E. coli* > *P. putida* > *B. subtilis* = *S. aureus* = *P. aeruginosa* while for the stems oil, the order is: *S. aureus* = *E. coli* > *P. putida* = *P. aeruginosa = B. subtilis.*

In general, the essential oil showed better antimicrobial activity against the Gram-positive bacteria than the Gram-negative bacteria. Gram-positive bacteria, *B. subtilis* and *S. aureus*, were the most susceptible to this oil, with inhibition zone of 7–12 mm with MIC values of 250–500 μg/mL. Gram-negative bacteria were most resistant to this oil with inhibition zones between 7–8 mm with MIC values of 125–500 μg/mL. These results are consistent to those of previous reports in the literature, indicating that Gram-negative bacteria are more resistant to essential oils than Gram-positive bacteria due to its outer membrane [30]. Some studies also concluded that the essential oils have greater antimicrobial activity due to their additive, synergistic or antagonistic effects [31].

## 3. Experimental Section

### 3.1. Plant Materials

Samples of *Piper caninum* Bl. were collected from Kuala Kangsar, Perak, Malaysia, in April 2011. This species was identified by Mr. Ahmed Zainuddin Ibrahim and the voucher specimen (HTBP3389) was deposited at the Herbarium of Taman Botani, Putrajaya.

### 3.2. Solvent and Chemicals

β-Carotene, linoleic acid, 1,1-diphenyl-2-picrylhydrazyl (DPPH), gallic acid and butylated hydroxytoluene (BHT) were obtained from Sigma-Aldrich Chemie (Steinheim, Germany). Analytical grade methanol, ethanol and dimethylsulfoxide (DMSO), HPLC grade chloroform, Folin-Ciocalteu’s reagent, anhydrous sodium sulfate, sodium carbonate, polyoxyethylene sorbitan monopalmitate (Tween-40) were purchased from Merck (Darmstadt, Germany).

### 3.3. Extraction of Essential Oils

The fresh leaves and stems were subjected to hydrodistillation in an all glass Dean-stark apparatus for 8 hours. The oils were dried over anhydrous magnesium sulfate and stored at 4–6 °C. The oils yields (w/w) were 0.46% and 0.31% for leaves and stems, respectively, based on their fresh weight.

### 3.4. Gas Chromatography (GC)

GC analysis were performed on a Hewlett Packard 6890 series II A gas chromatograph equipped with an Ultra-1 column (25 m long, 0.33 μm thickness and 0.20 mm inner diameter). Helium was used as a carrier gas at a flow rate of 0.7 mL/min. Injector and detector temperature were set at 250 and 280 °C, respectively. Oven temperature was kept at 50 °C, then gradually raised to 280 °C at 5 °C/min and finally held isothermally for 15 min. Diluted samples (1/100 in diethyl ether, v/v) of 1.0 μL were injected manually (split ratio 50:1). The injection was repeated three times and the peak area percents were reported as means ± SD of triplicates. Calculation of peak area percentage was carried out by using the GC HP Chemstation software (Agilent Technologies).

### 3.5. Gas Chromatography-Mass Spectrometry (GC-MS)

GC-MS chromatograms were recorded using a Hewlett Packard Model 5890A gas chromatography and a Hewlett Packard Model 5989A mass spectrometer. The GC was equipped with Ultra-1 column (25 m long, 0.33 μm thickness and 0.20 mm inner diameter). Helium was used as carrier gas at flow rate of 1 mL/min. Injector temperature was 250 °C. Oven temperature was programmed from 50 °C (5 min hold) to 250 °C at 10 °C/min and finally held isothermally for 15 min. For GC-MS detection, an electron ionization system, with ionization energy of 70 eV was used. A scan rate of 0.5 s (cycle time: 0.2 s) was applied, covering a mass range from 50–400 amu.

### 3.6. Identification of Constituents

The constituents of the oils were identified by comparison of their mass spectra with reference spectra in the computer library (Wiley) and also by comparing their retention indices, with those of authentic compounds or data in the literature [32]. The quantitative data were obtained electronically from FID area percentage without the use of correction factor.

### 3.7. Antioxidant Activity

#### 3.7.1. β-Carotene-Linoleic acid Assay

The β-carotene-linoleic acid bleaching assay described by Miraliakbari, and Shahidi [33] was used with minor modifications. A mixture of β-carotene and linoleic acid was prepared by adding together of 0.5 mg β-carotene in 1 mL chloroform (HPLC grade), 25 μL linoleic acid and 200 mg Tween 40. The chloroform was then completely evaporated under vacuum and 100 mL of oxygenated distilled water was subsequently added to the residue and mixed gently to form a clear yellowish emulsion. The essential oils (2 g/L) were in methanol and 350 μL of each sample solution were added to 2.5 mL of the above mixture in test tubes and mixed thoroughly. The test tubes were incubated in a water bath at 50 °C for 2 h together with two blanks, one contained BHT (positive control) and the other contained the same volume of methanol. The absorbance was measured at 470 nm on an ultraviolet-visible (UV–Vis) spectrometer. Antioxidant activities (inhibitions percentage, I%) of the samples were calculated using the following Equation:

(1) $$\left[\text{I}\%=(A_{\beta -\text{carotene after }2\;\text{h}}/A_{\text{initial }\beta -\text{carotene}})\times 100\right]$$

where *A*_β-carotene after 2 h_ assay is the absorbance value of β-carotene after 2 h assay remaining in the samples and *A*_initial β-carotene_ is the absorbance value of β-carotene at the beginning of the experiment. All tests were carried out in triplicate and inhibition percentages were reported as means ± SD of triplicates.

#### 3.7.2. DPPH Radical Scavenging Assay

The free radical scavenging activity was measured by the DPPH method as described by Loo, Jain, and Darah [34] with minor modification. Each sample of stock solution (1.0 mg/L was diluted to final concentration of 1000, 500, 250, 125, 62.5, 31.3, 15.63 and 7.81 μg/mL. Then, a total of 3.8 ml of 50 μM DPPH methanolic solution was added to 0.2 mL of each sample solution and allowed to react at room temperature for 30 min. The absorbance of the mixtures was measured at 517 nm. A control was prepared without sample or standard and measured immediately at 0 min. Lower absorbance of the reaction mixture indicates higher free radical scavenging activity, and vice versa. Inhibitions of DPPH radical in percent (I%) were calculated as follow:

(2) $$\left[\%=[(A_{\text{blank}}-A_{\text{sample)}})/A_{\text{blank}}]\times 100\right]$$

where *A*_blank_ is the absorbance value of the control reaction (containing all reagents except the test compound) and *A*_sample_ is the absorbance values of the test compounds. The sample concentration that provides 50% inhibition (IC_50_) was calculated by plotting inhibition percentages against concentrations of the sample. All tests were carried out in triplicate and IC_50_ values were reported as means ± SD of triplicates.

#### 3.7.3. Total Phenolic Content

Total phenolic contents of the essential oils were determined as described by Loo, Jain, and Darah [34]. Sample of stock solution (1.0 mg/mL) was diluted in methanol to final concentrations of 1000, 800, 600, 400, and 200 μg/mL. A 0.1 mL aliquot of sample was pipetted into a test tube containing 0.9 mL of methanol, then 0.05 mL Folin-Ciocalteu’s reagent was added, and the flask was thoroughly shaken. After 3 min, 0.5 mL of 5% Na_2_CO_3_ solution was added and the mixture was allowed to stand for 2 h with intermittent shaking. Then, 2.5 mL of methanol was added and left to stand in the dark for 1 h. The absorbance measurements were recorded at 765 nm. The same procedure was repeated for the standard gallic acid solutions and a standard curve obtained with the following Equation:

(3) $$\left[y=0.0021x-0.0223,r^{2}=0.9928\right]$$

The concentration of total phenolic compounds in the oils was expressed as mg of gallic acid equivalent per gram of sample. Test was carried out in triplicate and gallic acid equivalent value was reported as mean ± SD of triplicate.

### 3.8. Antimicrobial Activity

#### 3.8.1. Microbial Strains

The test microorganisms, *Staphylococcus aureus* (ATCC29737), *Bacillus subtilis* (ATCC6633), *Pseudomonas aeruginosa* (ATCC9027), *Pseudomonas putida* (ATCC49128), *Escherichia coli* (ATCC10536), *Candida albicans* (ATCC10231), and *Aspergillus niger* (ATCC16888) were used. The strains were grown on Nutrient agar (Oxoid, Italy) for the bacteria and Potato dextrose agar (PDA) for yeasts and fungi.

#### 3.8.2. Disc Diffusion Assay

Antimicrobial activity of the essential oils of *P. caninum* was carried out by the disc diffusion method reported by Murray, Baron, Pfaller, Tenover, and Yolken [35]. The essential oils were dissolved in DMSO (4 mg/mL). Inocolumn of 400 μL suspension containing 10^8^ CFU/mL of bacteria and 10^6^ CFU/mL of fungi each was spread on the nutrient agar (NA) and potato dextrose agar (PDA) medium. The discs (6 mm diameter) impregnated with 10 μL of the essential oils and DMSO (negative control) were placed on the inoculated agar, and were incubated for 24 h at 37 °C (bacterial), 48 h at 30 °C (yeast) and 72 h at 30 °C (fungi). Streptomysin sulfate (10 μg/mL) and nystatin (100 IU) were used as the positive controls for bacteria and fungi, respectively. Clear inhibition zones around the discs indicated the positive antimicrobial activity. All tests and analysis were carried out in triplicates.

#### 3.8.3. Minimum Inhibitory Concentration (MIC)

The minimal inhibitory concentration (MIC) was determined by broth micro dilution method using 96-well microplates as described by Gulluce [36]. The inocula of the microbial strains were prepared from 24 h broth cultures and suspensions were adjusted to 0.5 McFarland standard turbidity. Essential oil (1 mg) was dissolved in DMSO (1 mL) to get 1000 μg/mL stock solution. A number of wells were reserved in each plate for positive and negative controls. Sterile broth (100 μL) was added to well from row B to H. The stock solutions of samples (100 μL) were added to wells at row A and B. Then, the mixture of samples and sterile broth (100 μL) at row B were transferred to each well in order to obtain a twofold serial dilution of the stock samples (concentration of 1000, 500, 250, 125, 62.5, 31.3, 15.63 and 7.81 μg/mL). The inoculated bacteria (100 μL) were added to each well. The final volume in each well was 200 μL. Strepyomysin sulfate and nystatin were used as positive controls for bacterial and fungal, respectively. Plates were incubated at 37 °C for 24 h. Microbial growth was indicated by the turbidity and the presence of pellet at the bottom of the well.

### 3.9. Statistical Analysis

Data obtained from essential oil analysis, antioxidant and antimicrobial activity were expressed as mean values. The statistical analyses were carried out employing one way ANOVA (*p* < 0.05). A statistical package (*SPSS version 11.0*) was used for the data analysis.

## 4. Conclusions

The result demonstrated that safrole, β-caryophyllene, β-pinene and germacrene D were the main components from the leaves and stems oil of *P. caninum*. The highest activity was observed for inhibition of lipid peroxidation in the β-carotene/linoleic acid system by the stems oil. The essential oil of *P. caninum* showed strong antimicrobial activity; therefore it might well be used as an antimicrobial agent, as well as in food preservatives.

## Figures and Tables

**Table 1:** Components identified in the leaves and stems oils of *P. caninum*.

Components	KI b	Percentage a	

		Leaves	Stems
α-Pinene	932	4.0 ± 0.15	1.6 ± 0.08
Camphene	946	0.2 ± 0.14	0.6 ± 0.12
**β-Pinene**	974	**8.9 ± 0.09**	4.9 ± 0.11
Myrcene	988	0.9 ± 0.10	−
Limonene	1024	3.9 ± 0.18	2.7 ± 0.08
(*Z*)-β-Ocimene	1032	3.4 ± 0.06	0.2 ± 0.12
γ-Terpinene	1054	0.1 ± 0.09	−
α-Terpinolene	1086	0.4 ± 0.17	−
2-Nonanone	1087	1.2 ± 0.05	−
**Linalool**	1095	**7.0 ± 0.07**	2.9 ± 0.15
Camphor	1141	−	0.3 ± 0.08
Pinocarvone	1160	0.1 ± 0.15	−
Terpinen-4-ol	1174	0.2 ± 0.12	0.5 ± 0.05
α-Terpineol	1186	0.3 ± 0.10	1.0 ± 0.14
*n*-Decanal	1201	0.2 ± 0.15	0.8 ± 0.12
**Safrole**	1285	**17.1 ± 0.07**	**25.5 ± 0.07**
2-Undecanone	1293	2.0 ± 0.14	1.1 ± 0.12
δ-Elemene	1335	1.8 ± 0.11	4.1 ± 0.16
α-Cubebene	1345	−	0.5 ± 0.15
Eugenol	1356	−	2.4 ± 0.08
Cyclosativene	1369	0.2 ± 0.14	−
α-Ylangene	1373	0.2 ± 0.04	−
α-Copaene	1374	0.5 ± 0.06	0.9 ± 0.06
β-Cubebene	1387	−	0.3 ± 0.12
β-Bourbonene	1387	1.1 ± 0.08	
β-Elemene	1389	2.1 ± 0.14	2.4 ± 0.14
α-Gurjunene	1409	0.3 ± 0.08	0.9 ± 0.08
**β-Caryophyllene**	1417	**6.7 ± 0.12**	**9.8 ± 0.09**
β-Gurjunene	1431	0.2 ± 0.04	−
Aromadendrene	1439	−	0.8 ± 0.15
α-Humulene	1452	1.0 ± 0.13	1.6 ± 0.12
*allo*-Aromadendrene	1458	−	0.3 ± 0.07
**Germacrene D**	1484	4.9 ± 0.12	**7.8 ± 0.14**
Zingiberene	1493	0.4 ± 0.07	0.6 ± 0.21
Bicyclogermacrene	1502	1.1 ± 0.04	2.3 ± 0.14
(*E*,*E*)-α-Farnesene	1505	0.9 ± 0.08	−
α-Bisabolene	1506	−	0.4 ± 0.08
δ-Cadinene	1522	0.4 ± 0.04	1.0 ± 0.05
Chavibetol	1524	0.9 ± 0.11	−
Elemol	1548	0.2 ± 0.14	−
Germacrene B	1559	−	1.1 ± 0.11
(*E*)-Nerolidol	1561	3.9 ± 0.09	1.6 ± 0.17
Caryophyllene oxide	1582	0.2 ± 0.12	0.4 ± 0.07
Globulol	1590	−	0.3 ± 0.12
*t*-Muurolol	1644	−	2.4 ± 0.09
β-Eudesmol	1649	0.9 ± 0.06	−
α-Cadinol	1652	−	1.0 ± 0.06
Farnesyl acetate	1845	−	1.2 ± 0.15
**Group components**			
Phenylpropanoids		18.0 ± 0.21	27.9 ± 0.22
Monoterpene Hydrocarbons		21.8 ± 0.19	10.0 ± 0.18
Oxygenated Monoterpenes		7.5 ± 0.24	4.7 ± 0.16
Sesquiterpene Hydrocarbons		21.9 ± 0.18	35.2 ± 0.29
Oxygenated Sesquiterpenes		5.2 ± 0.21	6.9 ± 0.18
Others		3.5 ± 0.24	2.3 ± 0.12
Identified Components (%)		77.9 ± 0.24	87.0 ± 0.26

a Each value is expressed as means ± SD of three injections;

b Retention indices on Ultra-1 capillary column.

**Table 2:** Antioxidant activity of the leaves and stems oils of *P. caninum* a.

Samples	β-carotene/linoleic acid (%)	DPPH IC_50_ (mg/mL)	Total phenolic content Gallic acid equivalent (mg GA/g)
Leaves oil	103.5 ± 0.35	187.6 ± 0.45	27.4 ± 0.54
Stems oil	114.9 ± 0.42	452.4 ± 0.55	19.8 ± 0.42
BHT	95.5 ± 0.30	43.5 ± 0.25	ND

a Data represent mean ± standard deviation of three independent experiments; ND—not determined.

**Table 3:** Antimicrobial activity of the leaves and stems oils of *P. caninum* a.

	Leaves oil		Stems oil		Antibiotics SS		Nystatin	

Test microorganisms	DD b	MIC c	DD	MIC	DD	MIC	DD	MIC
*Bacillus subtilis*	12.2 ± 0.4	250	10.4 ± 0.5	250	17.6 ± 0.2	7.81	ND	ND
*Staphylococcus aureus*	7.2 ± 0.4	250	7.0 ± 0.5	125	17.8 ± 0.2	7.81	ND	ND
*Pseudomonas aeruginosa*	8.2 ± 0.4	250	8.8 ± 0.4	250	17.2 ± 0.2	7.81	ND	ND
*Pseudomonas putida*	7.8 ± 0.5	125	7.5 ± 0.4	250	17.3 ± 0.2	7.81	ND	ND
*Escherichia coli*	7.2 ± 0.4	62.5	7.8 ± 0.5	125	17.5 ± 0.2	7.81	ND	ND
*Candida albicans*	7.0 ± 0.3	>1000	7.8 ± 0.3	500	ND	ND	15.2 ± 0.2	7.81
*Aspergillus niger*	7.5 ± 0.3	>1000	8.0 ± 0.3	500	ND	ND	15.3 ± 0.2	7.81

a Data represent mean ± standard deviation of three independent experiments;

b DD—disc diffusion method (including the diameter of disc 6 mm);

c MIC—minimum inhibitory concentration (μg/mL);

SS—streptomysin sulfate; ND—not determined.
